# Patterns of grey matter loss associated with motor subscores in early Parkinson's disease

**DOI:** 10.1016/j.nicl.2017.11.009

**Published:** 2017-11-10

**Authors:** Xingfeng Li, Yue Xing, Antonio Martin-Bastida, Paola Piccini, Dorothee P. Auer

**Affiliations:** a Radiological Sciences, Division of Clinical Neuroscience, University of Nottingham, Queen's Medical Centre, Nottingham NG7 2UH, UK; b Sir Peter Mansfield Imaging Centre, School of Medicine, University of Nottingham, Nottingham NG7 2UH, UK; c NIHR Nottingham Biomedical Research Centre, Nottingham NG7 2UH, UK; d Centre for Neurodegeneration and Neuroinflammation, Division of Brain Sciences, Imperial College London, London W12 0NN, UK

**Keywords:** Parkinson's disease, MDS-UPDRS III, Motor symptoms, MRI grey matter, PD subtypes

## Abstract

Classical motor symptoms of Parkinson's disease (PD) such as tremor, rigidity, bradykinesia, and axial symptoms are graded in the Movement Disorders Society Unified Parkinson's Disease Rating Scale (MDS-UPDRS) III. It is yet to be ascertained whether parkinsonian motor symptoms are associated with different anatomical patterns of neurodegeneration as reflected by brain grey matter (GM) alteration. This study aimed to investigate associations between motor subscores and brain GM at voxel level. High resolution structural MRI T1 scans from the Parkinson's Progression Markers Initiative (PPMI) repository were employed to estimate brain GM intensity of PD subjects. Correlations between GM intensity and total MDS-UPDRS III and its four subscores were computed. The total MDS-UPDRS III score was significantly negatively correlated bilaterally with putamen and caudate GM density. Lower anterior striatal GM intensity was significantly associated with higher rigidity subscores, whereas left-sided anterior striatal and precentral cortical GM reduction were correlated with severity of axial symptoms. No significant morphometric associations were demonstrated for tremor subscores. In conclusion, we provide evidence for neuroanatomical patterns underpinning motor symptoms in early PD.

## Highlights

• MDS-UPDRS III score is inversely correlated with putamen and caudate grey matter intensity in patients with early Parkinson's disease (PD).
• Motor subscores reveal partially overlapping patterns of negative correlation with grey matter intensity for rigidity and axial symptoms.
• These findings point to early anterior striatal grey matter reduction underpinning the development of rigidity.
• Tremor scores are unrelated to grey mater morphometric changes.

## 1 Introduction

Parkinson's disease (PD) is the second most common neurodegenerative disorder characterized by rigidity, tremor, bradykinesia and loss of postural stability (Gibb, 1988). There is however, significant heterogeneity in the clinical presentation and course of the disease (Thenganatt and Jankovic, 2014, van Rooden et al., 2011, Marras and Chaudhuri, 2016). A number of MRI brain imaging methods (Brooks, 2010, Garg et al., 2015, Pyatigorskaya et al., 2014, Politis, 2014) have been applied to study the grey matter (GM) and white matter changes, and their association with clinical features of PD (Cochrane and Ebmeier, 2013, Gattellaro et al., 2009, Koshimori et al., 2015). GM density loss (Burton et al., 2004, Nagano-Saito et al., 2005, Beyer et al., 2007, Melzer et al., 2012, Koshimori et al., 2015) and atrophy (Melzer et al., 2012, Rosenberg-Katz et al., 2013, Mak et al., 2015, Delgado-Alvarado et al., 2016) in PD patients have been extensively studied using voxel-based morphometric methods. In contrast, relatively little attention has been paid to study the relation between MRI GM intensity changes and clinical motor measures as assessed with the Movement Disorder Society Unified Parkinson's Disease Rating Scale (MDS-UPDRS) III (Goetz et al., 2007). This is important because analysis of morphometric association of GM with clinical (sub)domain scores is a logical and powerful method to identify functionally meaningful brain structural patterns that may inform on PD biotypes.

Previous findings for the correlation between GM structural changes and MDS-UPDRS III were inconsistent. For example, one study based on brain image segmentation and cortical surface reconstruction found that there was a significant negative correlation between the MDS-UPDRS III score and the volume of the left caudate, but not with cortical thickness (Zarei et al., 2013). However, another study (Apostolova et al., 2010) did not show significant associations between MDS-UPDRS III subscale scores and caudate radial distance mapping an intuitive measure of the cortical thickness. Nevertheless, there are also several potential drawbacks with these earlier studies as they used relatively small sets of data (< 100 PD subjects). Moreover, none of the studies looked at the correlation between MDS-UPDRS III score and MRI GM intensity. This is likely to have limited the studies' sensitivity as previous experiments (Rosenberg-Katz et al., 2013, Mak et al., 2015, Delgado-Alvarado et al., 2016) showed that GM intensity was an arguably more reliable approach to investigate subcortical atrophy. Although significant correlation between GM concentration in the right middle frontal gyrus and a previous version of the UPDRS III score (Fahn and Elton, 1987) was observed (Melzer et al., 2012), MRI GM intensity associations with MDS-UPDRS III motor subscores have not been attempted. It thus remains to be shown which of the MDS-UPDRS III subscales best reflects symptoms arising from specific patterns of GM deficit. This would be an important step toward better understanding links between progression of the GM changes and clinical progression in PD (Vingerhoets et al., 1997). In this study, a T1 weighted sequence was adopted as the most popular method used to study brain structural changes in neurodegenerative disorders. It offers detailed, validated structural information including cortical thickness and GM density, which can inform on structural macroscopic disease effects and their patterns, and has been extensively used for PD morphometric studies (Pan et al., 2012). Diffusion tensor imaging (DTI) is also commonly used to study neurodegenerative disease based on its detailed characterisation of subtle white matter changes, but it is less well established to study neurodegenerative GM changes.

The purpose of this study was twofold. First, we used voxel-based correlational analyses to investigate the correlation between GM density and MDS-UPDRS III scores and its four main subscores (tremor, rigidity, bradykinesia, and axial symptoms) (Berganzo et al., 2016). This allowed us to identify regional patterns of neurodegeneration underpinning specific motor symptoms based on our hypothesis that GM intensity reduction may be a suitable index of early neurodegenerative pathology leading to reduced neuronal density before overt atrophic volume reduction. We postulated that mapping motor domains separately would reveal distinct GM patterns pointing to potential neural biotypes in PD.

Second, to test for potential confounding age effects we undertook repeat regression analysis controlling for age but limited to the striatum based on widely documented structural and diffusional alterations of the striatal nuclei in PD (Péran et al., 2010, Fioravanti et al., 2015). We hypothesised that there would not have significant age and striatal GM intensity correlation in PD.

## 2 Materials and methods

### 2.1 MRI dataset

Three hundred and ninety-two PD MRI T1 structural images were initially used in this study. All MRI image data were obtained from the PPMI website (http://www.ppmi-info.org/) on 13/10/2015 as previously published (Li et al., 2017). Also, the UPRDS III scores for these subjects were obtained from the PPMI database. Diagnosis of PD was performed by movement disorder specialists according to the PD UK Brain Bank Criteria. This excluded atypical Parkinsonism, concomitant vascular load, history of cognitive impairment, psychiatric disorders or other neurodegenerative disorders other than PD and any factors that would preclude MRI scanning. Tremor, rigidity, bradykinesia, and axial subscores were calculated according to the table in the supplementary material section (Supplementary Table). Table 1 shows the demographics and clinical details of the data used in the study.

**Table 1:** Demographics and clinical details of 364 subjects with PD (235 male) derived from PPMI repository.

	PD (mean ± SD)
Age	62.12 ± 9.77
Total MDS-UPDRS III score	21.63 ± 9.85
Sum of axial subscore	2.08 ± 1.48
Sum of bradykinesia subscore	9.68 ± 5.56
Sum of rigidity subscore	4.12 ± 2.89
Sum of tremor subscore	5.76 ± 3.42
Hoehn and Yahr Stage score	1.62 ± 0.52
Duration of disease (month)	6.70 ± 6.65
Number of patients on PD medication	117

SD: Standard deviation.

### 2.2 MDS-UPDRS III subscores

We divided the MDS-UPDRS III score into 4 subgroups according to motor symptoms in PD (Berganzo et al., 2016, Jankovic, 2008). MDS-UPDRS III scores were then subdivided into tremor (sum of items 15–18), rigidity (item 3), bradykinesia (sum of items 2, 4–9 and 14) and axial (sum of items 1 and 9–13) (Supplementary_Table). Group means of the total UPDRS III scores and the sums of items of the 4 subscores are displayed in Table 1.

### 2.3 Software packages for MRI data analysis

In the present study we employed several software packages/languages. The FSL-VBM package (http://fsl.fmrib.ox.ac.uk/fsl/fslwiki/FSLVBM) was adopted for image registration, image segmentation, GM modulation and image smoothing. The image registration toolkit (IRTK) (https://github.com/BioMedIA/IRTK) was also applied for image registration (Rueckert et al., 1999) if FSL failed for the image registration. MATLAB (www.mathworks.com) was used for MRI GM correlation analysis. In addition, Python language (https://www.python.org) was implemented to extract patient information from the clinical table, including age and total MDS-UPDRS III scores and subscores. To help localize GM differences, the 120 regions specified in the Automated Anatomical Labeling (AAL) template (Tzourio-Mazoyer et al., 2002) were used to label regions in the resultant statistical maps. Visual inspection was carried out at each step of image and clinical data processing.

### 2.4 MRI GM image processing

The MRI GM image was obtained using the FSL software package. First, structural T1 images were registered to the Montreal Neurological Institute (MNI) template using the FSL Linear Image Registration Tool (FLIRT) (Jenkinson and Smith, 2001) function. If the images failed to be registered, then the IRTK package with a manual registration was carried out to obtain the initial value for a rigid registration. A large head mask, as part of the MNI template, was employed to exclude shoulder and neck in the PPMI T1 brain image. This was done by multiplying the registered images with the head mask using FSL-maths functions. The Brain Extract Tool (BET) method (Smith, 2002) was employed to extract the brain (removing the skull from the whole image) for each of the 392 image sessions. Next, non-uniformity correction was carried out, and the FSL Automated Segmentation Tool (FAST v.4) (Zhang et al., 2001) was adopted to segment tissues according to their type. The segmented GM partial volume images were then aligned to the MNI standard space (MNI152) by applying the affine registration tool FLIRT (FMRIB's linear image registration tool) and nonlinear registration FNIRT (FMRIB's nonlinear image registration tool) methods, which use a B-spline representation of the registration warp field. The registered images (before smoothing), were averaged to create a study specific template, and the native GM images were then nonlinearly re-registered to the template image. The registered GM partial volume images were then modulated (to correct for local expansion or contraction) by dividing them by the Jacobian of the warp field. The segmented and modulated images were then smoothed with an isotropic Gaussian kernel with a standard deviation (sigma = 3 mm), and the final smoothed image (with sigma = 3 mm) was employed for the correlation analysis between brain GM and MDS-UPDRS III scores.

### 2.5 Correlation analysis and statistical inference

For GM and MDS-UPDRS III correlation analyses, 26 subjects were removed from the study due to GM segmentation problems, and two subjects had to be excluded due to missing MDS-UPDRS III scores resulting in a final dataset of 364 (235 male) subjects with PD (Table 1). We did not include a control group GM correlation analysis as the focus was on the interrelations with motor symptoms not present in control groups. For most healthy controls, the UPDRS III (sub)scores are 0 or a small number. It is mathematically easy to calculate, but difficult to interpret the correlation between the GM and UPDRS III score in healthy controls. Also, it is biologically not meaningful as these scores define the presence and the severity of symptoms rather than the degree of loss of normal function (Li et al., 2017).

We selected the MDS-UPDRS III scores collected from PPMI database nearest to the MRI scan time. Pearson correlation was calculated between MDS-UPDRS III and the smoothed GM image voxel by voxel. Then the correlation coefficient (r value) was converted to Z scores and T statistics for statistical inference. For the purpose of threshold correction, we also converted the T values to P values using MATLAB *tcdf*.*m* function. Based on P values and using FSL with family wise error (FWE) correction, we corrected the threshold for statistical inference for MDS-UPDRS III score/subscore and GM intensity correlation analysis.

To control for putative age and sex effects that may confound the symptom and GM associations, we applied a multivariate general linear model (GLM) to study the independent relationship between UPDRS III scores and GM intensities. We employed GM intensity as a dependent variable, and included total UPDRS III score, age, and sex as independent variables in the GLM:

(1) Y=a+b⋅UPDRS3+c⋅Age+d⋅Sex+e

where *Y* is GM intensity for each voxel, *a*, *b*, *c*, *d* are the regression coefficients and *e* is the model error. Applying the least square method to solve Eq. (1), we obtained the regression coefficient *c* for the age effect, and then we tested the significance of the coefficients.

This analysis was run after applying a striatal GM mask created from the AAL template. FSL-FWE method was then applied to determine the small volume corrected P < 0.05 threshold.

## 3 Results

### 3.1 Correlation between MDS-UPDRS part III total and subscores

To understand the mutual correlation between different MDS-UPDRS III subscores, we computed the cross correlation between these items (Table 2). We averaged the subscores of each class, and calculated the correlation between different classes. Although all the items in the table are significantly correlated with each other (P < 0.05, r > 0.1028), the tremor subscores show the weakest correlation strength with the other three subscores (considered very weak for all except rigidity (Evans, 1996)). The severity of tremor was less dependent on the severity of the other motor subscores, suggesting it could belong to a different biological subclass. The strongest interrelation was seen for bradykinesia, which was strongly correlated with rigidity, and moderately with axial symptoms. It should also be noted that bradykinesia displayed a very strong correlation with the total MDS-UPDRS III score (explaining about 79% of its variance), highlighting its cardinal role in PD and the MDS-UPDRS III score analysis.

**Table 2:** MDS-UPDRS III and its subscore cross-correlation matrix. 1st row/column: axial; 2nd row/column: bradykinesia; 3rd row/column: rigidity; 4th row/column: tremor; 5th row/column: average MDS-UPDRS III score (r > 0.1028, P < 0.05, degree of freedom is 362).

Correlation coefficient	Axial	Bradykinesia	Rigidity	Tremor	Average UPDRS III
Axial	1.0000	0.4850	0.3885	0.1031	0.5740
Bradykinesia		1.0000	0.6181	0.1985	0.8879
Rigidity			1.0000	0.2040	0.7715
Tremor				1.0000	0.5351
Total UPDRS III					1.0000

### 3.2 Association pattern of motor severity and GM intensity

**Fig. 1:**
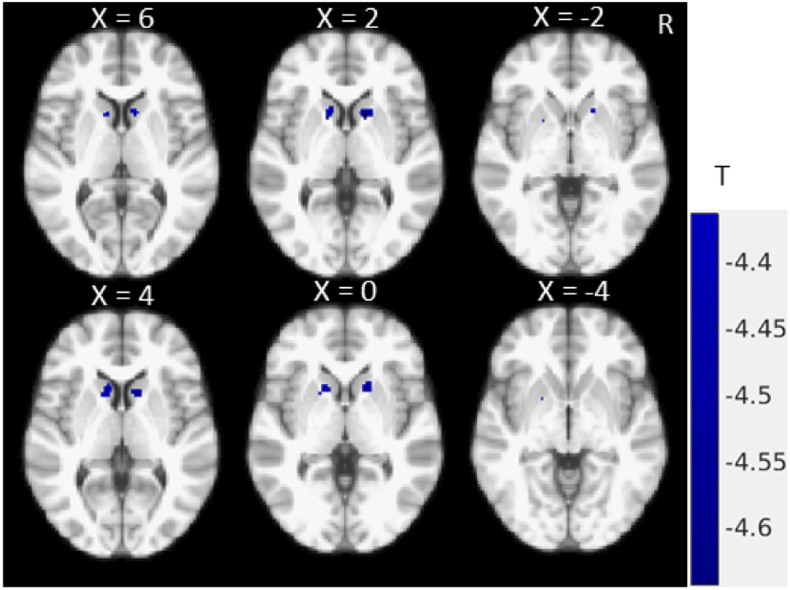
Correlation map showing significant negative associations between total MDS-UPDRS III score and striatal GM intensity (P < 0.05, FWE corrected).

Voxelwise correlation analysis revealed a significant negative association GM intensity and severity of total UPDRS III score (Fig. 1). Patients with more severe motor symptoms were found to have significantly less GM density in the heads of the caudate nuclei (MNI coordinate: (± 13.9, 14, 4), T = − 4.5) and the right anterior putamen (MNI coordinate: (15.8, 11.1, 0), T = − 4.42). No positive correlations were identified at P < 0.05 with FWE correction.

**Fig. 2:**
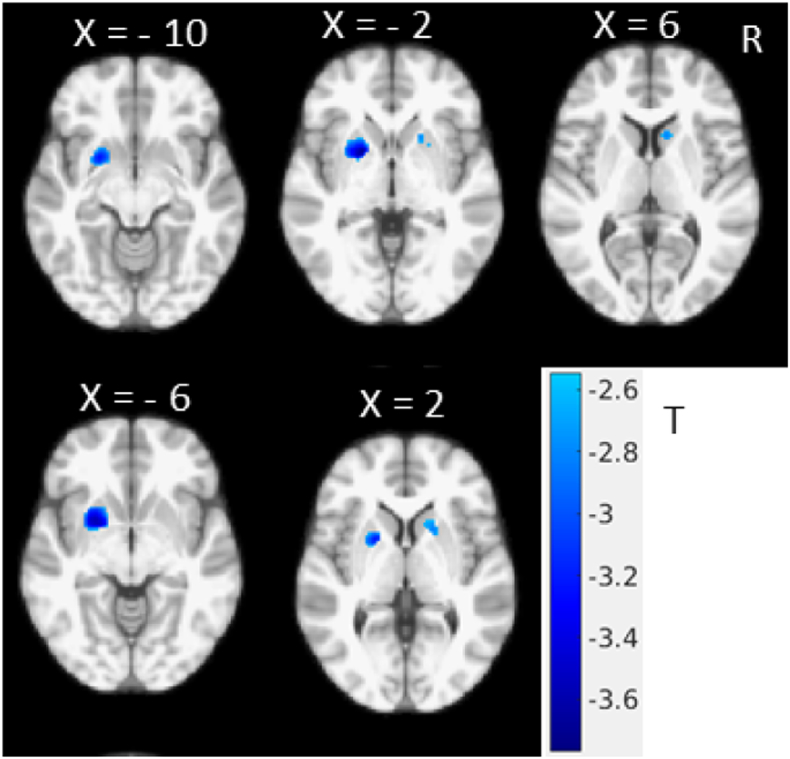
Multivariate regression model showing independent association between average MDS- UPDRS III score and GM density, considering age and sex as a co-variates in the model (T < − 2.548, one tailed, P < 0.05, FWE corrected, with small volume correction in striatum region). (For interpretation of the references to color in this figure, the reader is referred to the web version of this article.)

We studied the total MDS-UPDRS III score correlation with GM in a multivariate regression model taking age and sex effects into account (Eq. (1)). In the regression model, we tested coefficient *b* to quantify the correlation between MDS-UPDRS III and GM intensity. The colour regions in Fig. 2 show the significant (P < 0.05, FWE corrected threshold) associations in putamen and caudate regions.

Potential regional age effects were then further studied by testing the coefficient *c* in Eq. (1). We found that the regions with significant age effects were located mainly in the amygdala-hippocampus and parahippocampus regions (data not shown). We did not find significant age correlations with GM in other brain regions, in particular not the striatal regions, providing further support that our observed striatal GM motor association pattern is not affected by age.

Next, we investigated the strength of correlation by selecting significant clusters from Fig. 1 (putamen and caudate) as regions of interest for post hoc correlation analysis. Cluster-averaged GM intensities were then correlated with respective MDS-UPDRS III (sub-)scores. This showed significant but weak correlations (Table 3) between the GM intensity in the caudate and putamen with global motor severity (MDS-UPDRS III) and three of its subscores (axial, bradykinesia and rigidity). There was no significant correlation between striatal GM intensity and tremor severity.

**Table 3:** Correlation between GM intensity in caudate and putamen and MDS-UPDRS III scores. Bold and underlining indicate significant (P < 0.05, degree of freedom is 362) correlation.

Correlation coefficient	Axial	Bradykinesia	Rigidity	Tremor	Average UPDRS III
Caudate	−** 0**.**2195**	−** 0**.**1970**	−** 0**.**2354**	− 0.0718	−** 0**.**2382**
Putamen	−** 0**.**2353**	−** 0**.**2103**	−** 0**.**2363**	− 0.0493	−** 0**.**2405**

### 3.3 Association patterns of motor subdomains and GM intensity

**Fig. 3:**
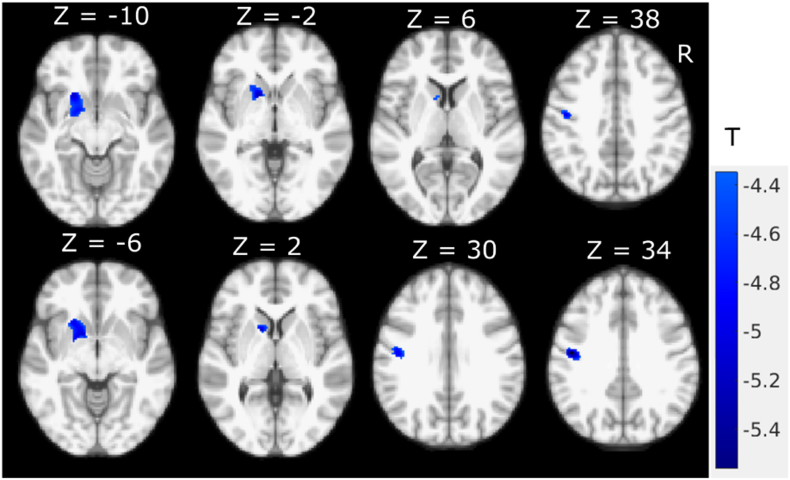
Correlation map between MDS-UPDRS-III axial motor subscores severity and GM intensity (P < 0.05, FWE corrected).

To further assess potential motor domain specific GM change patterns we used separate voxel-wise regression analysis for the motor subscores. Axial symptoms showed significant (P < 0.05, FWE corrected) negative correlations with left-hemispheric GM intensity (Fig. 3). In addition to the left putamen (MNI coordinate: (− 18.6, 13, − 6.6), T = − 4.87) and left caudate (MNI coordinate: (− 10, 12.1, 3.9), T = − 4.57), significant negative correlation was found between axial subscores and GM intensities in the left primary motor (BA4) (MNI coordinate: (− 46.3, − 10.8, 32.4), T = − 5.3) and pre-motor dorsal areas (BA6) (MNI coordinate: (− 49.2, − 6.1, 32.9), T = − 4.58).

**Fig. 4:**
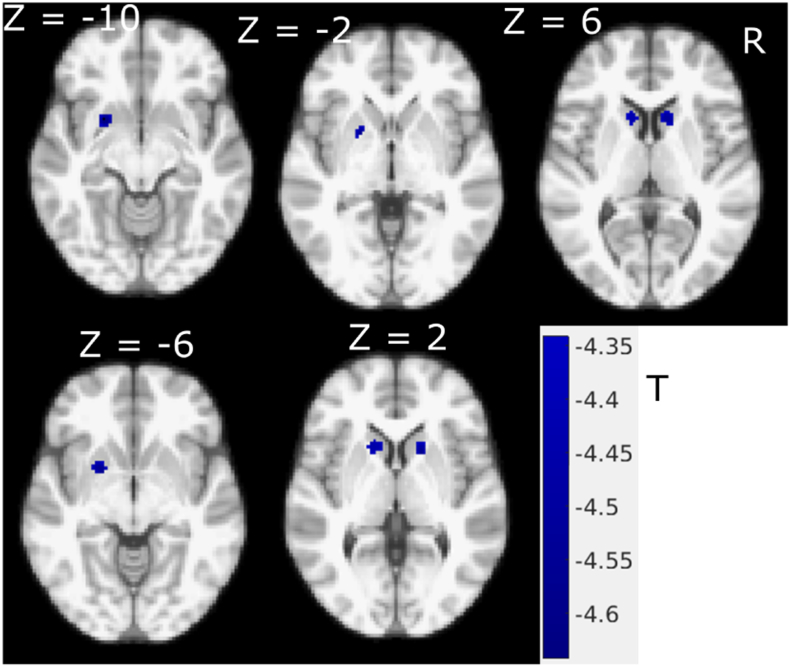
Rigidity subscores correlation with GM intensity in PD, coloured regions show significant negative correlation with FWE correction (P < 0.05). (For interpretation of the references to color in this figure, the reader is referred to the web version of this article.)

Similarly, correlation between the rigidity subscores and GM intensity showed a negative correlation in the putamen (MNI coordinate: (− 20.5, 10.2, − 4.5), T = − 4.37) and caudate (MNI coordinate: (± 12.9, 15.9, 3), T = − 4.48) (Fig. 4). The correlational pattern of GM and rigidity was qualitatively very similar to that of the MDS-UPDRS III total score (Fig. 1).

Correlation between GM and bradykinesia subscores was also calculated, revealing negative correlations in the putamen and caudate, which was not significant at the FWE corrected P < 0.05 level. Furthermore, voxel wise correlations did not show significant interrelations between tremor subscores and GM intensity at the corrected level, although we found significant negative correlations between severity of tremor and bilateral precentral cortical GM intensity (with FWE correction, there was no significant difference yet, P < 0.05). We also explored the interrelation between putamen and caudate regional Striatal Binding Ratio (SBR) of DATScan SPECT images as provided by the PPMI database with MDS-UPDRS III scores. No significant clinical correlation was found.

## 4 Discussion

In this study, we investigated the relationship between MRI GM intensity and MDS-UPDRS III scores and motor subscores. Fig. 1, Fig. 2 show the negative correlation between total UPDRS III score and GM intensity, and the apparent difference between Fig. 1, Fig. 2 is largely due to the addition of small volume correction for the multivariate analysis while the main findings and pattern remain remarkably similar suggesting a negligible age effect. We found an inverse association in the putamen and caudate demonstrating that increased total motor severity (MDS-UPDRS III) was linked with decreased anterior striatal GM density. Similar inverse association patterns were found for rigidity (MDS-UPDRS III subscores), while axial subscores showed negative correlation with the left hemispheric GM including precentral cortical areas (Fig. 3). With the FWE threshold correction, we only found significant left hemispheric correlations for axial motor scores. This could be due to the fact that the GM shows slight asymmetry in this dataset, with larger GM intensity in the right hemisphere. However, at more liberal (uncorrected, data not shown) threshold, both hemispheres show correlation with the axial motor subscore. Neither bradykinesia nor tremor scores were found to be significantly correlated with focal GM densities.

### 4.1 Comparing with previous studies

Although conventional structural MRI has been extensively used in PD to study GM loss and cortical atrophy (Burton et al., 2004, Pitcher et al., 2012, Camicioli et al., 2003), this is the first imaging study which used GM from a structural image to evaluate anatomical associations of motor symptoms in PD. Our structural MRI results show differences from and similarities with previous MRI and nuclear imaging (PET/SPECT) studies.

Our study contradicts a recent morphometric study (Garg et al., 2015), which showed no significant associations between GM changes and UPRDS III using MRI surface displacements information. Surface displacement captures disease related changes in the shape of the subcortical structures. This could be owing to the fact that the surface displacements were obtained from an image registration method that is sensitive to scale related changes (Garg et al., 2015). Our results also differ from other MRI studies (Apostolova et al., 2010, Zarei et al., 2013), which found absence of correlations (Apostolova et al., 2010) or between left caudate volume (Zarei et al., 2013) and motor severity (MDS-UPDRS III). This could be due to the methodological differences or reflect true differences in patient populations. Interestingly, we observed a left-sided predominance in the association pattern with axial symptoms specifically affecting the left caudate, which is partially in line with Zarei et al. (Zarei et al., 2013).

Our results are in good agreement with SPECT/PET studies with ^18^Fluorodopa (F-dopa) and dopamine transporter (DAT) tracers. F-dopa and DAT studies have traditionally been used to evaluate the disease severity of PD by assessing the integrity of dopaminergic terminals (Morrish et al., 1996, Puñal-Riobóo et al., 2009, Heiss and Hilker, 2004, Rahmim et al., 2016). PET/SPECT studies demonstrated negative correlation between MDS-UPDRS motor score and F-dopa and DAT concentration in caudate and putamen regions (Müller et al., 2000, Ziebell et al., 2010, Appel et al., 2015). The available data on the PPMI repository did not allow to undertake a voxel-based analysis in this cohort of patients and may well explain the lack to observe correlations between motor severity and regionally averaged SBR.

### 4.2 Relation with PD subtypes

Our GM results, however, suggest that functionally relevant changes of dopaminergic terminals in PD are co-localised and potentially underpinned by GM reductions. The observed association of GM intensity reduction with increasing motor severity in this early cohort of PD patients further points to early neural tissue damage in the anterior striatum, in keeping with neostriatal alpha synuclein pathology detected in stage III (Mori et al., 2008), and dendritic degeneration of medium spiny neurons (Zaja-Milatovic et al., 2005).

Interestingly, we also found that different PD motor domains have different GM correlation patterns, suggesting different motor striatal degeneration patterns. We identified similar GM intensity reduction in the anterior striatum with increasing severity of axial symptoms and rigidity, and at post hoc analysis level for bradykinesia. Tremor subscores, however, showed no correlation with GM density while axial symptoms also showed precentral cortical associations. We thus provide neuroanatomical support for neural biotypes that may underlie clinical phenotypes. This is important as a theoretical background to further justify recent attempts to use MRI for PD sub-classification, motivated by the increasing interest to subgroup PD into biologically distinct subtypes that may pave the way for a precision medicine approach in developing more effective treatments. Many different subtype classification systems and analysis methods have been proposed (Marras and Lang, 2013, Marras and Chaudhuri, 2016), with the best established classification according to the expression of two main motor symptoms into tremor-dominant (TD), postural instability with gait disorders (PIGD) subtypes and an intermediate form (Rosenberg-Katz et al., 2013). A recent latent class model of clinical information also revealed three distinct clinical PD subtypes (Campbell et al., 2016), whereas a model-based cluster analysis using baseline data suggested there may be four (van Rooden et al., 2011) or even five subtypes (Lawton et al., 2015). To determine clinically meaningful PD classes, it is likely that a multidimensional approach is needed that combines factors previously used such as clinical symptoms, genetic factors with imaging features (brain atrophy, cortical thinning, or functional imaging characteristics) (Rosenberg-Katz et al., 2013, Jankovic et al., 1990, Fereshtehnejad et al., 2015, Uribe et al., 2016). Our study demonstrates that GM density maps have additional classification potential and highlight a principled approach for feature selection based on powerful symptom-structure correlations using recently released large repository data.

To study the extent of a possible confounding age effect, we included age and sex of each subject as covariates in an additional regression model (Fig. 2). The multivariate regression results showed similar putamen and caudate GM correlation patterns with motor severity scales as seen in the univariate analysis (Fig. 1), that remained significant using a striatal small volume correction mask.

### 4.3 Advantages and limitations

The advantage of this study is that we used a large PD MRI dataset in the analysis. Without big data, GM and clinical correlations, significant differences would not be detected due to the small degree of freedom in the statistical comparison. A second advantage of the dataset is the large proportion of unmediated subjects (67.8%) thereby reducing the risk of drug-induced brain morphometric changes. The major limitation is that these results were based on cross-sectional data allowing us only to infer any associations, while longitudinal data is needed to confirm whether disease progression induces GM changes. At the level of cortical GM densities, we did not find interrelations with motor symptoms. This does however not exclude the possible presence of cortical pathology at a microscopic level that may either not affect GM density or be unrelated to motor symptoms. We also cannot exclude that clinically relevant cortical GM density changes may be a later phenomenon in PD as our large PD cohort includes mostly de-novo volunteers (Table 1). Finally, further investigation of the relation between dopaminergic functional imaging would be needed to clarify the molecular underpinnings of the observed structural/motor associations in early PD.

## 5 Conclusion

In a large sample of early PD, we found decreasing striatal GM density with increasing MDS-UPDRS III motor severity suggesting early anterior striatal neurodegeneration. We also observed different association patterns between motor domains with distinct subcortical GM intensity loss underpinning rigidity, cortical and subcortical GM reductions with increased axial symptoms, but no associations with bradykinesia or tremor severity.

## Appendix A Supplementary data

Supplementary table: UPDRS-III Subscores Calculation Method. Image 1
