# Supplementary material for: Patterns of grey matter loss associated with motor subscores in early Parkinson's disease
Source: Neuroimage Clin. 2017 Nov 10;17:498–504. doi: 10.1016/j.nicl.2017.11.009 (PMC5700824; doi:10.1016/j.nicl.2017.11.009)
Supplement: Supplementary file 1 — Supplementary table: UPDRS-III Subscores Calculation Method. [file mmc1.docx]

Supplementary Table part 1: UPDRS III subscore in Parkinson's Progression Markers Initiative (PPMI) clinical database.

**PPMI UPDRS III motor items**

| UPDRS column number | Scores | PPMI Variable | Clinical info | |  |  |
| --- | --- | --- | --- | --- | --- | --- |
| 1 | 3.1 | NP3SPCH | Speech |  |  |  |
| 2 | 3.2 | NP3FACXP | Facial expression | |  |  |
| 3 | 3.3a | NP3RIGN | Rigidity_Neck | |  |  |
| 4 | 3.3b | NP3RIGRU | Rigidity_Right Upper Limb | | |  |
| 5 | 3.3c | NP3RIGLU | Rigidity_Left Upper Limb | | |  |
| 6 | 3.3d | PN3RIGRL | Rigidity_Right Lower Limb | | |  |
| 7 | 3.3e | NP3RIGLL | Rigidity_Left Lowere Limb | | |  |
| 8 | 3.4a | NP3FTAPR | Finger tapping_Right hand | | |  |
| 9 | 3.4b | NP3FTAPL | Finger tapping_Left hadn | | |  |
| 10 | 3.5a | NP3HMOVR | Hand movements_Right Hand | | |  |
| 11 | 3.5b | NP3HMOVL | Hand movements_Left Hand | | |  |
| 12 | 3.6a | NP3PRSPR | Pronation-supination movements_Right Hand | | | |
| 13 | 3.6b | NP3PRSPL | Pronation-supination movements_Left Hand | | | |
| 14 | 3.7a | NP3TTAPR | Toe tapping_Right Hand | | |  |
| 15 | 3.7b | NP3TTAPL | Toe tapping_Left Hand | | |  |
| 16 | 3.8a | NP3LGAGR | Leg agility_Right hand | | |  |
| 17 | 3.8b | NP3LGAGL | Leg agility_Left hand | | |  |
| 18 | 3.9 | NP3RISNG | Arising from chair | |  |  |
| 19 | 3.1 | NP3GAIT | Gait |  |  |  |
| 20 | 3.11 | NP3FRZGT | Freezing of gait | |  |  |
| 21 | 3.12 | NP3PSTBL | Postural stability | |  |  |
| 22 | 3.13 | NP3POSTR | Posture |  |  |  |
| 23 | 3.14 | NP3BRADY | Global spontaneity of movements | | | |
| 24 | 3.15a | NP3PTRMR | Postural tremor_Right hand | | |  |
| 25 | 3.15b | NP3PTRML | Postural tremor_Left hand | | |  |
| 26 | 3.16a | NP3KTRMR | Kinetic tremor_Right hand | | |  |
| 27 | 3.16b | NP3KTRML | Kinetic tremor_Left hand | | |  |
| 28 | 3.17a | NP3RTARU | Rest tremor amplitude_Right upper limb | | | |
| 29 | 3.17b | NP3RTALU | Rest tremor amplitude_Left upper limb | | | |
| 30 | 3.18c | NP3RTARL | Rest tremor amplitude_Right lower limb | | | |
| 31 | 3.18d | NP3RTALL | Rest tremor amplitude_Left lower limb | | | |
| 32 | 3.18e | NP3RTALJ | Rest tremor amplitude_Lip/jaw | | | |
| 33 | 3.19 | NP3RTCON | Constancy of rest | |  |  |

Supplementary Table part 2: tremor, bradykinesia, rigidity, and axial subscore calculation using supplementary Table part 1.

**NEW UPDRS-III (max = 132)**

**Tremor (max = 40)**

- Postural tremor of the hands X 2 (0-8)
- Kinetic tremor of the hands X 2 (0-8)
- Rest tremor amplitude - extremities, lip/jaw X 5 (0-20)
- Constancy of rest tremor (0-4)

**Bradykinesia (max = 52)**

- Facial expression (0-4)
- Finger tapping X 2 (0-8)
- Hand movements X 2 (0-8)
- Pronation-supination movements of hands (0-8)
- Toe tapping X 2 (0-8)
- Leg agility X 2 (0-8)
- Arising from chair (0-4)
- Global Spontaneity of movement (0-4)

**Rigidity (max = 20)**

- Rigidity each joint X 5 (0-20)

**Axial (max = 20)**

- Speech (0-4)
- Gait (0-4)
- Freezing of gait (0-4)
- Postural stability (0-4)
- Posture (0-4)
